# Epidemics of Community-Associated Methicillin-Resistant *Staphylococcus aureus* in the United States: A Meta-Analysis

**DOI:** 10.1371/journal.pone.0052722

**Published:** 2013-01-02

**Authors:** Vanja M. Dukic, Diane S. Lauderdale, Jocelyn Wilder, Robert S. Daum, Michael Z. David

**Affiliations:** 1 Department of Applied Mathematics, University of Colorado, Boulder, Colorado, United States of America; 2 Department of Health Studies, University of Chicago, Chicago, Illinois, United States of America; 3 Department of Pediatrics, University of Chicago, Chicago, Illinois, United States of America; 4 Department of Medicine, University of Chicago, Chicago, Illinois, United States of America; National Institutes of Health, United States of America

## Abstract

*Staphylococcus aureus* is the most frequent cause of skin and soft tissue infections in humans. Methicillin-resistant strains of *S. aureus* (MRSA) that emerged in the 1960s presented a relatively limited public health threat until the 1990s, when novel community-associated (CA-) MRSA strains began circulating. CA-MRSA infections are now common, resulting in serious and sometimes fatal infections in otherwise healthy people. Although some have suggested that there is an epidemic of CA-MRSA in the U.S., the origins, extent, and geographic variability of CA-MRSA infections are not known. We present a meta-analysis of published studies that included trend data from a single site or region, and derive summary epidemic curves of CA-MRSA spread over time. Our analysis reveals a dramatic increase in infections over the past two decades, with CA-MRSA strains now endemic at unprecedented levels in many US regions. This increase has not been geographically homogeneous, and appears to have occurred earlier in children than adults.

## Introduction

*Staphylococcus aureus* is among the most common bacterial pathogens of human beings and the most frequent cause of skin and soft tissue infections (SSTIs), osteomyelitis, and bacteremia [1]. Strains of *S. aureus* that are resistant to all ß-lactam antibiotics (with the exception of ceftaroline), known as methicillin-resistant *S. aureus* (MRSA), were first identified among hospitalized patients in 1960 [2]. For approximately three decades, until the late 1980s, MRSA remained a predominantly nosocomial infection. Beginning in the 1990s, however, new strains of community-associated (CA-) MRSA began to cause infections in previously healthy people in the U.S. [3]. CA-MRSA strains differ from the older healthcare-associated (HA-) MRSA isolates in several ways: they typically cause different clinical syndromes, infect different groups of patients, and are genetically distinct [4] from HA-MRSA strains. Since the early 1990s, CA-MRSA infections have become a common cause of infections in the general population of the U.S., with evidence of particularly high risk in household contacts of those with a MRSA infection, athletic facilities, nursing homes, kindergartens, and jails [5]. CA-MRSA infections are occasionally fatal in otherwise healthy people. Much about the extent of the CA-MRSA problem in the U.S. is unknown: when these strains first arose, how rapidly they have increased as a cause of infection, and whether the increase was similar across the country and for both children and adults.

National hospital surveillance programs have long tracked MRSA infections. By the mid-1990s, for example, more than half of *S. aureus* infections in U.S. intensive care units were caused by MRSA isolates [6], clearly establishing that MRSA had become an endemic problem and a threat to patients in hospitals. However, no such national or regional surveillance is in place for CA-MRSA. Invasive CA-MRSA infections are tracked by the Centers for Disease Control and Prevention’s (CDC’s) Active Bacterial Core Surveillance (ABC) network [7], but the great majority of CA-MRSA infections are not invasive and would not be captured by this surveillance program. CA-MRSA infections in the U.S. are not surveilled.

Understanding the epidemiology of CA-MRSA is crucial to establishing public health interventions to control MRSA. There have been many descriptive studies from single medical centers or individual cities on the epidemiology of MRSA, and they each document a rise in the number of infections caused by CA-MRSA in specific geographic location in the U.S. [5]. However, they address different years, different definitions of CA-MRSA, and different metrics to report MRSA incidence, making it difficult to extrapolate from them and to understand the national trend, or geographic variation across the U.S.

To overcome these challenges in comparing individual studies over time, we carried out a meta-analysis in which we identified studies that reported infection rates in two or more years from the same clinical or geographic location within the U.S. and then grouped together studies that used comparable metrics. We performed separate meta-analyses for each type of metric. These “meta-epidemic” analyses allow us to estimate the average year of origin of CA-MRSA, rate of increase, and the estimated year and level of the peak infection rates, both overall (for the entire U.S.) and individually for specific geographic locations. They document national trends of a rapid increase and more recent plateau in CA-MRSA infections.

## Materials and Methods

### Search Strategy and Selection Criteria

The studies we used for this meta-analysis were obtained by searching Medline for all citations on the epidemiology of MRSA (with keywords “MRSA”, “ORSA”, “methicillin-resistant *Staphylococcus aureus*”, “meticillin-resistant *Staphylococcus aureus*”, or “oxacillin-resistant *Staphylococcus aureus*” and the keyword “epidemiology”) published from January 1, 1990 to September 30, 2012. The search was limited to studies in English and MRSA. Among the 5052 citations identified, abstracts were reviewed for the inclusion criteria. Studies were included if they were performed in the U.S. and if they contained data on a rate of CA-MRSA infections (defined epidemiologically) from at least 2 time periods, spanning at least 12 months in duration, and in the same geographic location. Additional published articles were identified from citations in these publications. When published reports did not include actual data (i.e., data were presented only in a graph or summarized), we asked the corresponding author to share the underlying tabular data, and the reports were included if the author provided these data.

### Study Classification

Only studies that included both a count of CA-MRSA infections and a reference denominator for the count numerator were included. There were many different types of denominators reported in studies, but just three denominator types were used by multiple studies. These were: 1) studies of the incidence of CA-MRSA infections in a population, 2) studies of the proportion of all *S. aureus* infections that were CA-MRSA, and 3) studies of the proportion of all MRSA infections that were CA-MRSA.

### Statistical Analysis

Data on CA-MRSA temporal trends in the three study groups were used to perform three separate meta-analyses of the temporal variation in CA-MRSA across the U.S. These meta-analyses differ from classic meta-analysis that estimate a pooled treatment or exposure effect from a series of clinical trials or observational studies; instead our aim is to estimate a pooled temporal effect.

**Figure 1:**
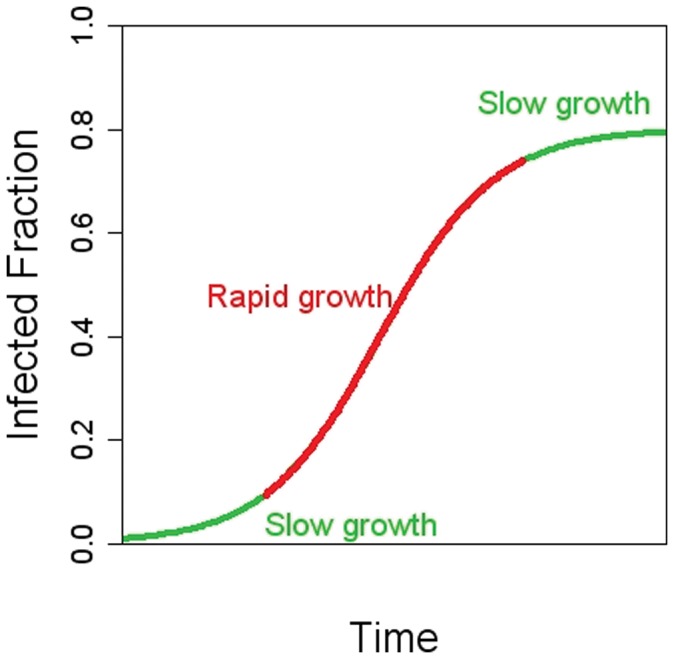
Illustration of the logistic curve in modeling the fraction of the infected people in a population over time. The infection rate is assumed to grow as a non-linear function of time. The growth rate is low when only a small fraction in a society is infected. After a certain period of time, when there are sufficiently many infections circulating in the population, the number of contacts between the susceptible people (those without the disease) and those with infection increases – as a result, a period of rapid growth of infections occurs. Eventually, the growth slows down again, and the fraction of infections levels off, settling at an endemic level as the time goes on.

To model the spread of CA-MRSA over time, we employed the three-parameter logistic growth curve model [8]. Logistic growth functions have a sigmoid shape, and are often encountered in population ecology and infectious disease epidemiology, where they are used to model non-linear growth in the number of the infections over time (the logistic growth curve model, in its differential equation form, corresponds to the “susceptible-infectious-susceptible” family of models). This model assumes that the increase in the number of infections in the population depends on the interaction between infected and non-infected individuals. This is a common principle in models of infectious disease spread – implying that the growth of the infected population will be slow either when the number of already infected people is small, or when the number of those remaining susceptible in the population is small. Please see Figure 1 for more details.

Note that while we use the terms “epidemic” and “epidemic curve” to describe the temporal trend in CA-MRSA infections, we do not have the classic outbreak data that follow an infection wave (increase in the number of cases followed by a decrease back to nominal levels). Instead, the logistic growth model is appropriate to describe the incidence of a new pathogen growing over time to a relatively stable, new, endemic level. Under the hypothesis that this model accurately describes the dissemination of CA-MRSA strains in the U.S. thus far, we can estimate the average rate of dissemination of the CA-MRSA epidemic, assess geographic differences in its emergence, and compare the dissemination of the new strains in children and adults.

The meta-analysis was performed using a fixed-effects approach in order to capture individual study differences due to potential unmeasured confounding factors (confounding the rate of spread), such as societal structure, access to health care, and environmental factors. The fixed-effect analysis produces a quantitative summary (pooled) meta-epidemic curve for the group of studies used in each meta-analysis, without assuming a structure for the underlying population of all possible studies, an assumption that a random-effect meta-analysis would make.

The resulting meta-curves were thus estimated using a fixed-effects meta-analytic model of all study-specific curves, fitted separately within each of the three groups of studies, as follows. Fixed study-specific intercepts were used to allow different times of onset of CA-MRSA epidemics in each location Similarly, study-specific growth rates were used to estimate how rapidly the rate of CA-MRSA grew in different study populations. Study-specific saturation factors were used to represent the endemic levels at which the CA-MRSA rates ultimately plateau.

### The Model

In the following logistic growth model:
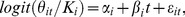
(1)the logit of the scaled θ_it_ (the CA-MRSA proportion reported by the study i in year t) is modeled as a linear function of time t (expressed in years since 1980). The rate of dissemination is modeled by the fixed study-specific effect β_i_, with the higher values of β corresponding to the faster spread of disease. In addition, each study’s epidemic curve can be shifted along the time axes, depending on when CA-MRSA infections first appeared. This is determined by the study-specific fixed parameter α_i_, with higher values of α corresponding to later emergence. The parameter K_i_ is the saturation level, corresponding to the peak CA-MRSA rate that is achieved over time in that population. The errors ε_it_ are assumed to be independent, and distributed normally with mean zero and a constant study-specific variance σ_i_^2^. The assumption of normally distributed errors was verified in residual analysis.

For each of the three groups of studies, we constructed a figure that includes all data points from each study from that group, the fitted curve for points from the same study, and, for the first two groups of studies, a meta-epidemic curve for all of the studies in the group. In addition, in the third set of studies (studies in which CA-MRSA is reported as a proportion of all MRSA infections), we separated studies of adult and pediatric populations. We focused on three features of these fitted curves: 1) the first year when the fraction crossed a small threshold (the value taken as the initial notable occurrence of CA-MRSA infections); 2) the peak rate level as an indicator of the endemic level; and 3) the year when the rate will approach the endemic level. The strength of fit for each curve was assessed by examining the correlation coefficient between observed and fitted points.

## Results

Seventeen studies summarizing CA-MRSA cases over time (using one of the three types of denominators) were identified. The studies were performed in ten U.S. states [9]–[22] and the data were collected from 1988 through 2009. A summary of the studies included is shown in Table 1.

**Table 1:** Summary of studies in the meta-analyses, grouped by the type of denominator in the rate.

Location	Years	Age Group	Population type	Infection type	Numerator	Denominator		Reference
**CA-MRSA/Population Count**								
Chicago, IL	2000–5	Adult	I/P, ED, O/P	SSTIs	CA-MRSA (modified CDC criteria)	100,000 pop. in catchmentarea		[9]
Maryland	1999–2008	Adult	I/P, O/P	All	CA-MRSA (48-hour criterion)	100,000 veterans		[10]
Tricare	2005–10	Ped/Adult	I/P, ED, O/P	SSTIs and bloodinfections	CA-MRSA (3 calendar-day criterion)	100,000 person-years		[11]
Pennsylvania	2005–9	Ped/Adult	I/P, O/P	All	CA-MRSA (CDC criteria)	100,000 person-years		[12]
**CA-MRSA/All ** ***S. aureus***								
Springfield, MA	2000–8	Ped	I/P, ED, O/P	SSTI requiring incisionand drainage	CA-MRSA (no comorbid conditions)	All *S. aureus* (no comorbid conditions)		[13]
Denver, CO	2002–4	Ped/Adult	I/P, ED, O/P	SSTIs	CA-MRSA (24–hour criterion)	All *S. aureus*		[14] *
Morristown, NJ	2003–7	Ped	ED	SSTIs	Any MRSA	All SSTIs		[15]
**CA-MRSA/All MRSA**								
Chicago, IL	1988–90, 1993–95, 1998–99, 2004–5	Ped	I/P	All	CA-MRSA	All MRSA	[3], [16]	
Corpus Christie, TX	1990–2003	Ped	I/P, ED, O/P	All	MRSA	All *S. aureus*	[15], [18] *	
Memphis, TN	2000–2	Ped	I/P, O/P?	All	CA-MRSA (CDC criteria)	All MRSA	[19]	
Minneapolis, MN	1991–2003	Ped	I/P, O/P	All	CA-MRSA (CDC criteria)	All MRSA	[20] *	
Minnesota	2000–5	Ped/Adult	I/P, O/P	All	CA-MRSA (CDC criteria)	All MRSA	[21]	
San Diego, CA	1994–97	Adult	I/P, O/P	All	CA-MRSA (24 hr criterion)	All MRSA	[22]	
St. Louis, MO	1996–2005	Adult	I/P, ED, O/P	All	non-MDR MRSA	All MRSA	[23]	
St. Louis, MO	2002–4	Adult	I/P	Invasive	non-MDR MRSA	All MRSA	[24]	
Northern CA	1998–2009	Ped/Adult	I/P, ED, O/P	All	CA-MRSA (modified CDC criteria)	All MRSA	[25] *	

* Summarized data used to create published figures was received from the corresponding authors of these studies. Notes: CDC stands for Centers for Disease Control and Prevention; I/P stands for inpatient; O/P stands for outpatient; ED stands for emergency department; CA-, stands for community-associated; MDR stands for multidrug resistant; MRSA stands for methicillin-resistant *Staphylococcus aureus*; Ped. stands for pediatric; Pop. stands for population; and SSTIs stands for skin and soft tissue infections. CDC, 24-hour, 48-hour, and 3-calendar-day criterion refer to different criteria defining CA-MRSA infections. The last 3 categorized infections as being CA-MRSA if they were cultured in an outpatient or cultured from a patient within the stated period after admission to a hospital.

### Population-based CA-MRSA Incidence

**Figure 2:**
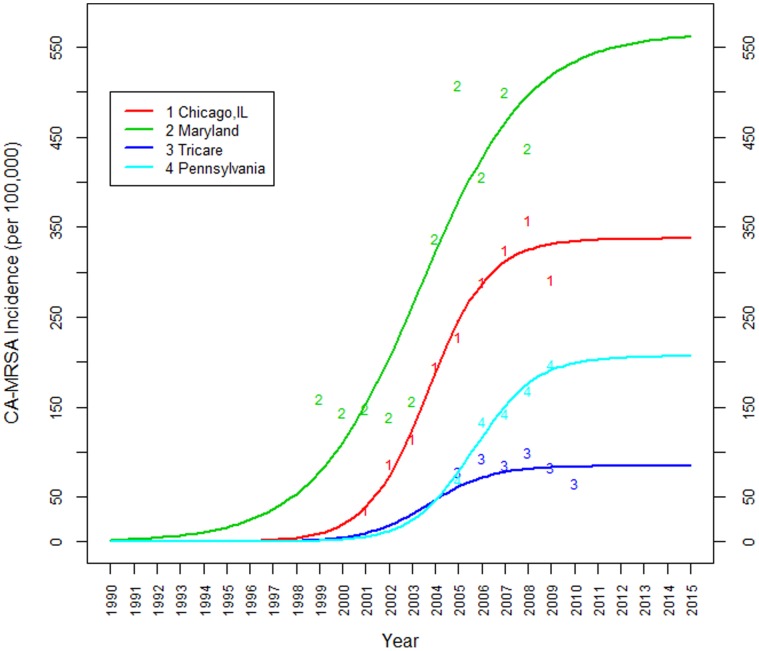
Estimated population incidence rate meta-curve, based on the two CA-MRSA population incidence studies from Chicago, IL [**9**], Maryland [**10**], the population insured under Tricare [**12**] and Pennsylvania [**11**]. Only marginal changes are expected after year 2010.

There were four studies reporting data from CA-MRSA cases as a fraction of a large population: (1) general population in Chicago [9] (2) population of military veterans in Maryland [10], (3) greater than 440,000 adult and pediatric patients in Pennsylvania who are served by the Geisinger Health System [11], and (4) the population across the U.S. insured under Tricare, which includes more than 9 million active duty military personnel, military retirees, certain reservists, and immediate family members [12]. The Chicago population is an extrapolated estimate based on patients seen in the public Cook County system of hospitals and clinics. The Chicago and Maryland data each include about ten years of observation, while the Tricare and Pennsylvania populations include five to six years. Figure 2 shows the individual rates, as well as the estimated epidemic curve for each of the studies. However, we chose not to present the meta-epidemic curve in this figure: due to significant differences among the four populations, the meta-curve’s interpretation as the average population curve becomes questionable.

Individually, the peak CA-MRSA incidence rate among Maryland veterans was estimated at 566 per 100,000 people (standard error: 180·5 per 100,000), Chicago at 337 per 100,000 people (standard error: 21 per 100,000), Tricare at 84 per 100,000 (standard error: 5 per 100,000) and Pennsylvania at 206 per 100,000 (standard error: 39·3 per 100,000). From Figure 2, it appears that the incidence has recently been approaching a plateau, and according to our analysis we expect only marginal increases in population incidence rates in these four populations after the year 2011.

Our results also show that the epidemic among Maryland veterans seems to have preceded the others by approximately 10 years. For example, the incidence rate among Maryland veterans is estimated to have crossed the threshold of 1 case per 1,000,000 people around the middle of 1983, while this happened in Chicago in 1993, Tricare in 1995, and Pennsylvania in 1996. The Chicago, Pennsylvania and Maryland epidemics also appear to have spread slightly faster than the Tricare epidemic, though the difference in the rate of dissemination is not statistically significant: the estimated growth coefficients were 0·75 (standard error: 0·15) in Chicago, 0·75 (standard error: 0·4) in Pennsylvania, 0·75 (standard error: 1) in Tricare, and 0·42 (standard error: 0·22) in Maryland.

### CA-MRSA as a Proportion of all *S. aureus* Infections

**Figure 3:**
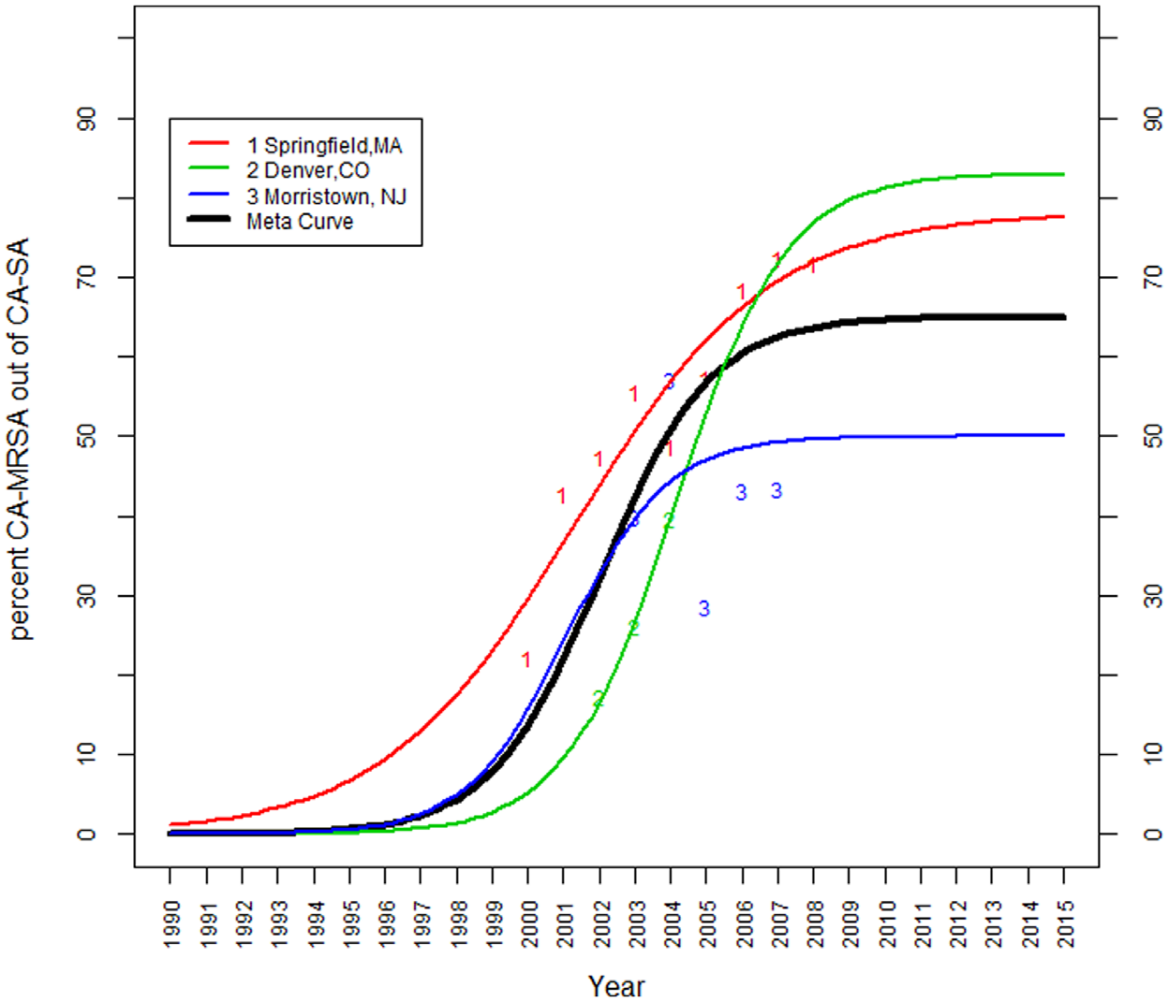
Estimated population meta-curve for studies reporting CA-MRSA as a proportion of all *S. aureus* infections. The estimated limiting fraction of CA-MRSA among all *S. aureus* infections is 65%. The individual studies vary with respect to this limiting fraction: in Springfield, MA [13], it was estimated at 78%, in Denver, CO [14] at 83% and in Morristown, NJ [15] at 50%.

Figure 3 shows the meta-epidemic curve estimated from three studies reporting CA-MRSA as a proportion of all *S. aureus* infections, along with the three individual fitted epidemic curves describing the CA-MRSA fraction over time in each study. The studies, performed at medical centers in Springfield, MA [13], Denver, CO [14], and Morristown, NJ [15], were found to vary significantly with respect to both the average fraction of CA-MRSA among all *S. aureus* (ranging from 28% to 54% of infections), as well as with respect to the estimated plateau: the peak proportion of CA-MRSA among all *S. aureus* infections in the study from Denver, CO was estimated at 83%, in Morristown, NJ at 50%, and in Springfield, MA at 78%. Based on these results, the meta-analytic plateau for the fraction of CA-MRSA out of all *S. aureus* infections is estimated to be 65% (standard error: 11%).

Our meta-analysis results indicate that the proportion of CA-MRSA among all *S. aureus* infections seems to have already stabilized, and is currently staying close to its plateau level, at all sites. Thus, minimal increase in the proportion of CA-MRSA among all *S. aureus* cases is predicted beyond 2010. Based on the estimates of origin, it appears that the first CA-MRSA cases in Springfield, MA preceded Denver, CO and Morristown, NJ by approximately six years. Based on our model, the percent of CA-MRSA among all *S. aureus* infections in Springfield, MA crossed the threshold of 0·01% in 1984, while this happened in Denver, CO in 1991, and Morristown, NJ in 1990. Furthermore, the rate of increase in the fraction of CA-MRSA among all *S. aureus* infections over time appears to be similar in Denver, CO and Morristown, NJ – the estimated growth coefficient is 0·65 in Denver, CO, and 0·71 in Morristown, NJ. The growth coefficient for Springfield, MA was estimated at a lower value of 0·37, implying slightly slower growth in the fraction of *S. aureus* infections that were CA-MRSA. However, the differences among the three growth coefficients are not statistically significant.

### CA-MRSA as a Proportion of all MRSA Infections at a Medical Center

**Figure 4:**
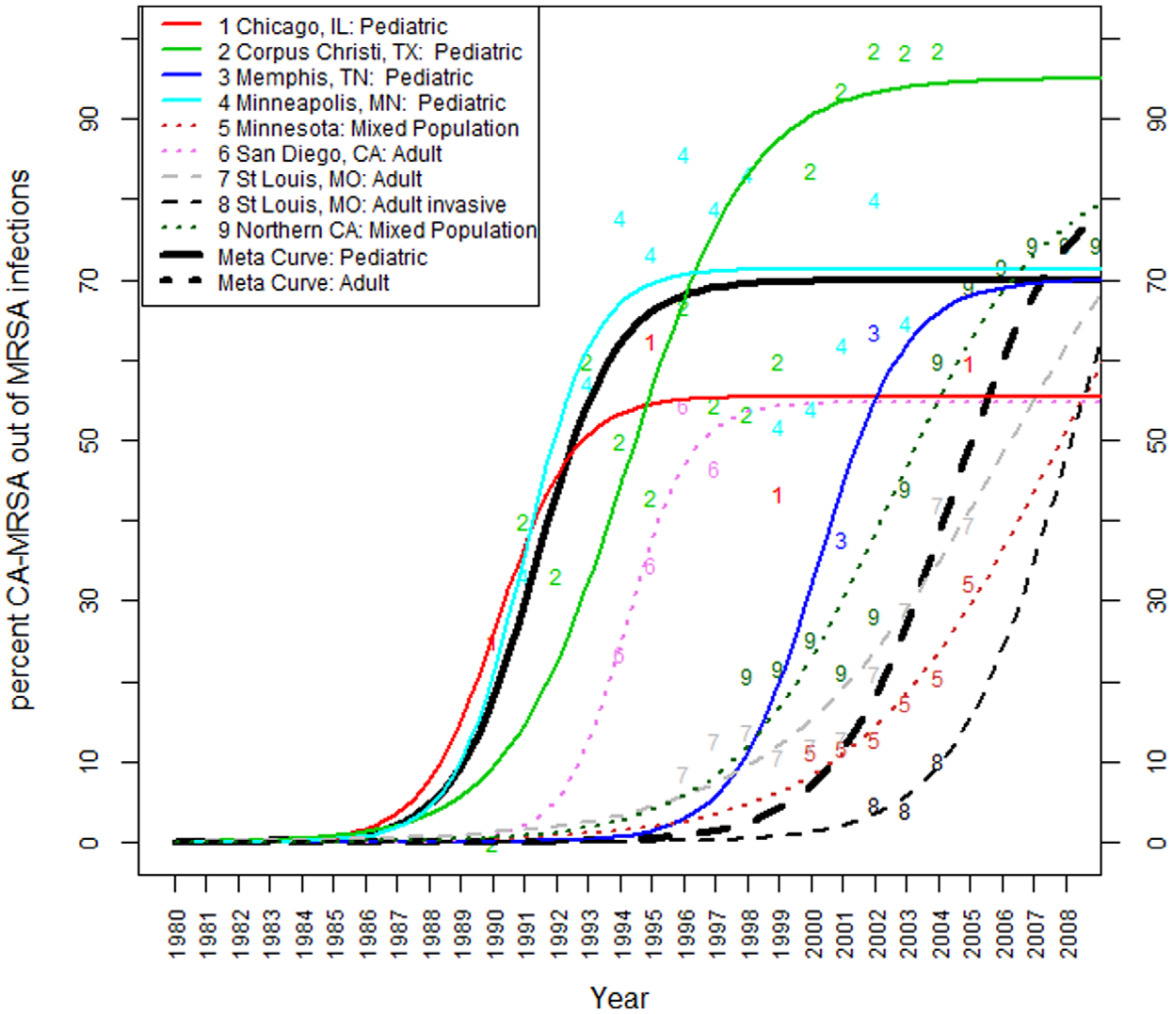
Estimated meta-curves for studies reporting CA-MRSA as a proportion of all MRSA infections at a medical center, estimated based on: Chicago, IL data from two studies [**3**], [**16**] and our data from the University of Chicago Medical Center; Corpus Christie, TX [**17**]–[**18**]; Memphis TN [**19**]; Minneapolis, MN [**20**]; Minnesota [**21**]; San Diego, CA [**22**]; two St. Louis studies [**23]**–[**24**]; and a study from Northern California [**25**]. The average pediatric CA-MRSA fraction began leveling off in about 1995, coming close to the plateau rate of 69·9%. The fraction of CA-MRSA among adult and mixed adult and pediatric MRSA cases seems to be still on the rise in almost all centers, except for the two mixed populations in San Diego, CA and Northern California. The fraction of CA-MRSA among adult MRSA cases in all centers seems to be continuing an upward trend. Our analysis predicts further increases of CA-MRSA’s share of all adult MRSA infections beyond 2010; the model estimates that the average long-term plateau of CA-MRSA to MRSA ratio among adults will be about 80%, with large variation among individual studies (56·9% to 100%).

Figure 4 shows meta-epidemic curves estimated from ten studies reporting on CA-MRSA as a proportion of all MRSA infections at nine medical centers or populations [3], [16]–[25]. The pediatric and adult studies are shown in Figure 4 to facilitate comparisons. However, separate meta-epidemic curves are shown for the pediatric and adult populations, as pediatric data from each individual center suggest that the rise of CA-MRSA among children occurred earlier than among adults. Three mixed adult and pediatric studies [21], [22], [25] were included in the adult meta-analysis, as the temporal pattern from those studies was more similar to the adult group. However, the omission of these studies in a repeated analysis showed very little effect on the resulting estimate of the meta-epidemic curve for adults.

The CA-MRSA fraction of all MRSA infections at medical centers has historically been higher among children than among adults. Based on our results, CA-MRSA cases seem to have emerged about eight to nine years earlier among children; the average percent of CA-MRSA of all MRSA infections exceeded 0·1% in 1984 among children and in 1992 among adults. The rate of dissemination was also more rapid among children than among adults (the average pediatric growth coefficient was estimated at 0·79, while the average adult growth coefficient was estimated at 0·55), although this difference was not statistically significant.

The average CA-MRSA fraction (of all MRSA infections) among children appears to have stabilized around 1995. The plateau fraction of all CA-MRSA pediatric cases among all pediatric MRSA infections at a medical center or in a studied population, was estimated at 70% (standard error 3·9%), with the individual centers ranging from 55% of all MRSA pediatric cases in Chicago due to CA-MRSA, to 95% of all MRSA pediatric cases in Corpus Christi. Although there is substantial variability among medical centers and populations studied, all individual pediatric study epidemic trajectories seem to have already stabilized by 2010, with no significant increases predicted beyond 2010.

In contrast to pediatric CA-MRSA, the fraction of CA-MRSA among mixed and adult MRSA cases appears to still be on the rise at almost all medical centers. The exception is the study from San Diego, CA [20] which included active military personnel and their dependents, and appears to be following the early spread pattern of the pediatric studies. However, this study only reported data from early years of the epidemic (1995 and 1997), so uncertainty remains about the subsequent progression of the CA-MRSA to MRSA ratio for that study population.

Only one of the adult studies [25] reported data beyond 2005, and, only that study (Northern California mixed population) started showing signs of leveling off of the CA-MRSA fractions around 2007. The estimated average plateau of the percentage of CA-MRSA among all mixed and adult MRSA infections is estimated to reach about 83% by the year 2015, with notable variation among individual studies (56·9% to 97%), assuming the current trajectories continue. One study (Schramm et al. [24]) reported the CA-MRSA fraction among all invasive MRSA cases in St. Louis, MO. Because most CA-MRSA infections are not invasive, the results of Schramm et al., as expected, show a lower proportion than the other studies in this category, reporting CA-MRSA among all MRSA infections. However, the Schramm et al. study does suggest that the fraction of CA-MRSA among all invasive MRSA cases is also on the rise.

In terms of geographic comparisons, the earliest notable increase of CA-MRSA among MRSA infections was estimated to have occurred in adults in St Louis, MO [23], and slightly later in pediatric patients in Corpus Christi, TX [17]–[18], followed by the pediatric patients in Chicago, IL [3], [16]. The fraction of CA-MRSA among all MRSA infections is estimated to have exceeded 0·1% in 1981 in St. Louis adults, in 1982 in Corpus Christi children, and in 1983 in Chicago children. We estimate that this threshold was then crossed among pediatric cases in Minnesota [20] in 1984, in a mix of adult and pediatric cases in Minnesota [21] and in Northern California [25] in 1986, and in the mix of adult and pediatric cases in San Diego, CA [22] in 1987. The most recent of the pediatric series to cross the 0·1% threshold was Memphis, TN [19] in 1993. Finally, the rates in one study to examine CA-MRSA as a fraction of invasive adult MRSA cases (St. Louis, MO [24]) were estimated to have crossed the 0·1% threshold in 2000.

Though the range of the estimated rate of dissemination (growth coefficients) is fairly wide, from 0·27 in St Louis, MO [23] to 0·91 in Minnesota [20] these differences are not statistically significant; the data are consistent with the percent of MRSA infections that were CA-MRSA growing at a similar rate at all sites studied.

### Conclusions

Many single-center studies have demonstrated an increase in MRSA infections outside of the health care setting in the past two decades. Using all available published data in our models, we confirmed this dramatic increase across the U.S. in CA-MRSA infections, and for the first time, we documented a plateau in this trend that differs by age group. CA-MRSA infections now appear to be endemic and at unprecedented levels in many regions. This is true whether we measure CA-MRSA infections as a proportion of all *S. aureus* infections, as a proportion of all MRSA infections, or as population incidence. This increase has not been geographically homogeneous, and we found that the dissemination of CA-MRSA strains appears to have occurred earlier in children than among adults.

Our models suggest that CA-MRSA infections began to appear in the U.S. in the early or middle 1980s, earlier than most published studies would suggest, and increased slowly through the 1990s. Beginning around 2000, there was a rapid rise that started to plateau in the late 2000s – with the exception of pediatric cases which appeared and plateaued earlier. Cases among military populations also appeared and plateaued earlier than other adult populations. The rapid rise coincided with the recognition of USA300, now the predominant strain of CA-MRSA in the U.S. [5]. Overall, our models, largely based on urban populations, support the findings of previous studies that have shown that, with the emergence of CA-MRSA strains, there was a rapid, consistent, nation-wide shift in the genetic backgrounds of *S. aureus* strains causing human infections in the U.S. in a relatively short period of time. Our study does not address why this shift occurred; this remains a conundrum for further study.

Our findings contrast with those from the CDC’s ABC, which recorded only invasive MRSA infections, a clinically severe subset of MRSA infections diagnosed most often among hospitalized patients [7], [26]. The CDC’s ABC data demonstrate that the incidence of health care-associated invasive MRSA infections decreased steadily in 2004–2008 [26]; this may reflect interventions by infection control programs in the health care setting, or the changing genetic backgrounds of MRSA clones circulating in that setting. In the present study we found that the incidence of CA-MRSA infections rose dramatically among adults in the same period. This apparent inconsistency is plausible because CA-MRSA infections are predominantly non-invasive and tend to have their onset outside of the health care setting.

There are several limitations to our study. First, to perform the meta-analyses, we grouped together studies that were similar but not identical in case definition and denominator definition. While it was previously demonstrated that there is overlap in identifying CA-MRSA cases defined by various criteria (the CDC definition, 48-hour criterion, non MDR criterion, etc.) [27], differences among the three studies of CA-MRSA infections as a proportion of all *S. aureus* infections (Figure 3) may relate, in part, to differences in patient populations and case definitions. The case series in Morristown, NJ [15] and Springfield, MA [13] only included children while the study from Denver, CO [14] included both children and adults. Similarly, the New Jersey study was restricted to patients in the emergency department (ED) and the denominator was all SSTI ED patients; this was deemed appropriate as the vast majority of SSTIs in an ED are in fact caused by *S. aureus*. The three studies also use slightly different criteria to distinguish CA-MRSA and HA-MRSA. However, despite these differences, the curves have similar shape and time course.

Second, the studies in our meta-analyses are predominantly from cities, and they may not reflect the epidemiology of CA-MRSA in rural areas of the U.S., where a slower trajectory in the CA-MRSA epidemic has been reported [28]–[29]. However, it is worth noting that a surveillance study of ten microbiology laboratories in both rural and urban Minnesota in 1996–1998 [30] was consistent with the modeled curve shown for the Como-Sabetti et al. study [21] in Figure 4. Third, the earlier rise of CA-MRSA as a proportion of all MRSA in children compared with adults (see Figure 4) may reflect a true contrast with adults in the rate of CA-MRSA dissemination, or it may in part reflect the relative infrequency of HA-MRSA infections in children compared to adults. Fourth, our projection of the model into years beyond the published data in studies included in the meta-analyses does not account for the possibility that there may be subsequent changes in the molecular epidemiology of *S. aureus* that could affect the incidence of CA-MRSA infections. Any such change would require additional modeling features, and revised predictions. Fifth, for studies that used a non MDR bacterial phenotype as the criterion for CA-MRSA infections, it is possible that an increase over time in the number of resistant classes of antibiotics for USA300 MRSA would lead to a progressive rise in the underestimation of the percent of MRSA infections that were CA-MRSA. If this is a bias in the included studies, our curves would underestimate the increase in CA-MRSA infections relative to all MRSA infections. Our data nevertheless demonstrated an increasing incidence of CA-MRSA infections among all MRSA infections, suggesting that this trend likely reflects a true increase.

Sixth, there are several methodological limitations. It is important to point out that the model employed in this analysis, based on the logistic curve, is only an approximation of reality. While it was able to describe the changes in the epidemiology of CA-MRSA, we cannot be sure about its performance in the future. In particular, the leveling-off that we are seeing might not be permanent, and the future might in fact hold a decline in the CA-MRSA rates. In addition, we have described the course of CA-MRSA spread in several geographic regions and have noted the differences in that spread in different locations. Our model is not however a spatial model: it does not account for distance between different study centers, and cannot be used to estimate rates of CA-MRSA spread in locations outside of those analyzed in this paper. Finally, the fixed-effects approach we take in this paper corresponds to the limit of the random effects approach, obtained as the variances of the random effects approach infinity. While a fully random effect approach could be used instead, the number of studies available for this meta-analysis would make our results sensitive to any choice of the random effect variances. Due to a lack of any information about these variances, we only present the fixed effect results in this paper. Our meta-analysis in the second and third group of studies should thus be viewed only as an average CA-MRSA spread in individual subpopulations, and not as a “true epidemic curve” corresponding to the spread in the underlying meta-population.

There were several studies of incident CA-MRSA infections that did not meet the inclusion criteria for our meta-analysis, in particular the requirement that they include data from more than one year to estimate a site-specific trend. A few studies were excluded because they were the only study to use a particular type of denominator and therefore could not be grouped with any other studies for meta-analysis. In other cases we could not include a study because we were unable to obtain the data represented in published graphs. There were also several studies with only case counts and no denominator. The data from all these studies, however, were consistent with our meta-curves. For example, CA-MRSA incidence reported in two single-year population-based studies in California [31]–[32] are both quite similar to the rates of CA-MRSA infections in Chicago, IL [9] estimated from the fitted meta-curve in the corresponding years (the only city population incidence data represented in the meta-analyses). In addition, data from two studies in veterans’ populations [33]–[34] are consistent with the meta-curves in Figure 3, although shifted slightly to the right. Four pediatric studies that did not meet our inclusion criteria also support the trends shown in our meta-curves [35]–[38].

In summary, our analysis shows that in the U.S., CA-MRSA infections, including both invasive and noninvasive disease, likely first appeared in the 1980s, rose dramatically between the mid-1990s and 2005, and have recently leveled off. We found evidence of considerable geographic variation, with the timing of the first reported cases varying by more then ten years between locales. We also provide evidence that CA-MRSA infections reached a steady endemic level earlier as a proportion of all MRSA infections among children, then later, among adults. CA-MRSA infections appear to have reached a plateau around 60 to 70% of all MRSA infections among children as early as the middle 1990s, but this proportion was likely still rising among adults in 2010. Additional population-based studies of CA-MRSA infection incidence in large population groups are needed to confirm the stability of the plateau values estimated by our models for more recent years.
